# Student satisfaction with the Mawhiba classes program: a multi-dimensional analysis across gender and educational levels in Saudi Arabia

**DOI:** 10.3389/fpsyg.2026.1732626

**Published:** 2026-02-23

**Authors:** Fahad S. Alfaiz

**Affiliations:** Self-Development Skills Department, Common First Year Deanship, King Saud University, Riyadh, Saudi Arabia

**Keywords:** gifted education, Mawhiba classes program, Saudi Arabia, self-contained classrooms, student satisfaction

## Abstract

This study assessed student satisfaction with the Mawhiba Classes Program (MCP), a specialized educational initiative in Saudi Arabia designed to meet the needs of gifted students. Using a quantitative research approach, the study analyzed satisfaction levels of 1,403 students across elementary, middle, and high school levels. Participants were recruited utilizing stratified random sampling methodology. The assessment focused on three dimensions: registration procedures, learning and teaching, and teacher performance. Results showed high levels of satisfaction across all dimensions, with elementary school students reporting significantly higher satisfaction compared to middle and high school students. Gender differences were minimal but not statistically significant. These findings highlight the importance of understanding student perspectives in gifted education programs and suggest that developmental considerations should inform program design. This study contributes to the growing body of knowledge on gifted education satisfaction assessment by providing empirical evidence on student perceptions while identifying areas for program enhancement to better support gifted learners’ educational experiences.

## Introduction

Student satisfaction represents a critical indicator of educational program quality and effectiveness, particularly in specialized educational settings designed for gifted learners. The assessment of student satisfaction provides valuable insights into program strengths and areas for improvement, informing evidence-based decisions for program enhancement (Preckel et al., 2008). In the context of gifted education, understanding student perspectives becomes even more crucial, as these learners often have unique educational needs and expectations that differ from their typical peers (Neihart, 2007).

Self-contained gifted classrooms have emerged as one of the most prevalent models for serving gifted students, designed to provide specialized learning environments that address the unique cognitive, social, and emotional needs of this population. These classrooms group gifted students together for full-time instruction, allowing for accelerated pacing, advanced content, and specialized pedagogical approaches (VanTassel-Baska and Brown, 2007). The effectiveness of such programs is often evaluated through multiple lenses, including academic achievement outcomes, social–emotional development, and importantly, student satisfaction and engagement.

### What does research say about self-contained gifted classrooms?

Self-contained gifted classrooms, sometimes referred to simply as gifted classrooms, have shown mixed results regarding their effectiveness based on existing research. These classrooms can effectively address the academic needs of gifted students by offering differentiated curricula, accelerated pacing, and opportunities for intellectual collaboration with similarly capable peers (Vidergor and Gordon, 2015; Linn-Cohen and Hertzog, 2007; Feldhusen and Sayler, 1990). For instance, a study evaluating a full-time, self-contained gifted classroom found that participating students demonstrated significantly greater gains on general cognitive ability tests compared to control groups (VanTassel-Baska et al., 1989). Additionally, research indicates that gifted students placed in self-contained classrooms may develop more positive attitudes toward school and their own giftedness (Jambura, 2013).

Self-contained classrooms have been the focus of research to evaluate their impact on student achievement, particularly for gifted and disadvantaged students. Card and Giuliano (2014) found that placing students in separate gifted classrooms significantly improves reading and math achievement, especially for lower-income, Black, and Hispanic students. This approach is particularly beneficial for students who are often excluded from traditional gifted and talented programs. Their findings suggest that a separate classroom environment is especially effective for students selected based on prior achievement, addressing inequities faced by disadvantaged populations.

The effectiveness of self-contained classrooms can vary across different academic areas. McGrath and Rust’s (2002) study demonstrated that self-contained classrooms improved overall performance on the Tennessee Comprehensive Assessment Program. However, their findings revealed no significant differences in students’ outcomes in math, reading, or social studies, indicating that the benefits of self-contained classrooms may depend on specific contexts or subjects.

The perceptions of students, teachers, and parents provide valuable insights into the effectiveness of self-contained gifted classrooms. One study found that grouping gifted students together fosters positive attitudes by allowing them to interact with peers of similar abilities, while also enhancing their interest, curiosity, and engagement with learning. Parents reported satisfaction with their decision to transfer their children to these specialized classrooms, and teachers expressed appreciation for the opportunity to work with this unique population (Vidergor and Gordon, 2015). These findings underscore the importance of tailored learning environments for gifted students and the satisfaction of key stakeholders.

Grouping gifted students in specialized environments enhances their motivation to learn, reduces academic boredom, and fosters deeper intellectual growth (Vidergor and Gordon, 2015; Chessor, 2014). These settings allow students to engage meaningfully with peers of similar abilities, creating an environment that encourages intellectual collaboration and exploration.

Teachers in self-contained classrooms often implement personalized teaching strategies, such as integrated units, student choice, and enrichment activities, to meet the unique needs of gifted learners (Linn-Cohen and Hertzog, 2007; Feldhusen and Sayler, 1990). Educators report greater flexibility in adapting instruction to gifted students and designing lessons that emphasize advanced conceptual understanding. However, research highlights the necessity of specific training in advanced pedagogy and enrichment curriculum design for teachers working with this population. Rhey emphasized that teaching gifted students often leads to a shift toward reform-based teaching practices, particularly for topics requiring conceptual complexity (Çaylak and Çakıroğlu, 2024).

While self-contained classrooms provide a strong foundation for academic growth and effective teaching for gifted students, they also present socio-emotional and equity-related challenges. Some students report feelings of loneliness and limited opportunities to interact with diverse peers, which can impact their social development (Cash and Lin, 2021). Addressing these concerns requires improved teacher training, flexible grouping strategies, and supportive classroom practices to balance academic and social needs. To better understand the long-term impacts of self-contained classrooms, further longitudinal studies are needed. Such research could help identify strategies to support both the intellectual and socio-emotional development of gifted students while addressing issues of equity and inclusion in specialized educational settings.

Self-contained classrooms require substantial funding, teacher expertise, and structural commitment to sustain effective differentiation and meet the needs of gifted students (Feldhusen and Sayler, 1990; Yüreğilli et al., 2021). Additionally, significant equity issues persist, such as the underrepresentation of minority gifted students, which remains a major barrier to the fair and inclusive implementation of self-contained classrooms (Borders et al., 2014).

Some research suggests that separate classrooms are not essential for the success of gifted students. Studies indicate that separate gifted programs in elementary schools often fail to significantly improve academic achievement. Instead, alternative models, such as schoolwide enrichment programs or push-in services within inclusive classroom settings, have been proposed as more effective solutions (Potter and Burris, 2019). Other findings have demonstrated that gifted students can achieve at high levels in regular classroom environments when provided with appropriate differentiation, enrichment, and support (Taylor, 2003; Winner, 1996).

In conclusion, while self-contained classrooms may offer certain benefits for gifted students, effective teaching practices and meaningful differentiation can support high achievement in a variety of educational settings. The key lies in addressing the unique needs of gifted learners through appropriate challenges, sustained engagement, and equitable support, regardless of the classroom model.

### Student satisfaction in gifted education

Student satisfaction in educational contexts is grounded in several theoretical frameworks, including self-determination theory (Deci and Ryan, 2000) and expectancy-value theory (Eccles and Wigfield, 2002). Self-determination theory suggests that satisfaction is enhanced when students experience autonomy, competence, and relatedness in their learning environment. For gifted students, this translates to having choices in their learning, experiencing appropriate academic challenge, and connecting with intellectual peers (Renzulli, 2012).

Expectancy-value theory posits that student satisfaction is influenced by their expectations for success and the value they place on the educational experience. Gifted students often have high expectations for their educational experiences and value intellectual challenge, creativity, and meaningful learning opportunities (Reis and McCoach, 2000). When programs meet these expectations and provide valued experiences, student satisfaction increases.

### Factors influencing student satisfaction in gifted education

Research has identified several key factors that influence student satisfaction in gifted education programs. Academic challenge and appropriate pacing are fundamental components, as gifted students often experience boredom and disengagement in traditional classroom settings that do not provide sufficient intellectual stimulation (Kanevsky and Keighley, 2003). Self-contained gifted classrooms address this need by offering accelerated curricula and advanced content that matches students’ abilities and interests.

Teacher quality and expertise represent another critical factor in student satisfaction. Gifted students benefit from teachers who understand their unique characteristics and can implement specialized instructional strategies (VanTassel-Baska, 2018). Research indicates that students report higher satisfaction when their teachers demonstrate knowledge of gifted education principles, use differentiated instruction, and create supportive learning environments (Moon and Brighton, 2008).

Social factors also play a significant role in student satisfaction. Gifted students often struggle with social isolation and difficulty finding intellectual peers in traditional classroom settings (Margot and Melin, 2021). Self-contained classrooms provide opportunities for gifted students to interact with like-minded peers, fostering a sense of belonging and reducing feelings of isolation (Peterson, 2009).

Program organization and administrative factors, including registration procedures and program communication, also influence student satisfaction. Clear, efficient processes and transparent communication help create positive program experiences and reduce stress for students and families (Olszewski-Kubilius and Clarenbach, 2012).

### Gender differences in educational satisfaction

Research on gender differences in educational satisfaction has revealed complex patterns that vary by context and educational level. In gifted education specifically, studies have found that male and female students may have different preferences and satisfaction patterns related to various program components (Preckel et al., 2008). Some research suggests that female gifted students may place greater value on social connections and supportive teacher relationships, while male gifted students may prioritize academic challenge and competition (Kerr and McKay, 2013).

However, these patterns are not universal and may be influenced by cultural context, program design, and individual differences. In Middle Eastern educational contexts, cultural factors may further influence gender differences in educational satisfaction and program participation (Aljughaiman et al., 2009). Understanding these potential differences is important for ensuring that gifted education programs meet the needs of all students regardless of gender.

### Developmental changes in program satisfaction

Student satisfaction with educational programs often varies across developmental stages and grade levels. Research suggests that younger students tend to report higher satisfaction with school experiences, with satisfaction often declining during adolescence (Neihart, 2007). This pattern may be particularly pronounced in gifted education, where older students may develop more sophisticated expectations and become more critical of their educational experiences.

Elementary-aged gifted students often show high enthusiasm for specialized programs and may be less critical of program components (Robinson et al., 2021). Middle school students may experience increased social awareness and comparison with peers, potentially affecting their satisfaction with specialized programming (Margot and Melin, 2021). High school students may focus more on college preparation and future goals, influencing their evaluation of program components and overall satisfaction (Olszewski-Kubilius and Clarenbach, 2012).

### Cultural factors in student satisfaction assessment

The assessment of student satisfaction must consider cultural context, as cultural values and educational traditions influence student expectations and evaluation criteria. In the Saudi Arabian context, cultural emphasis on family involvement in education, respect for teachers, and academic achievement may influence how students evaluate their educational experiences (Aljughaiman et al., 2009).

Additionally, the collectivistic cultural orientation common in Middle Eastern societies may influence how students express satisfaction and dissatisfaction, potentially leading to more positive responses or reluctance to express criticism (Hofstede, 2001). Understanding these cultural factors is essential for accurate interpretation of satisfaction data and program evaluation.

### Gifted education in Saudi Arabia

The Kingdom of Saudi Arabia has made significant investments in gifted education through the establishment of the King Abdulaziz and His Companions Foundation for Giftedness and Creativity (Mawhiba) in 1999. The foundation’s mission is to identify, nurture, and support gifted individuals across the Kingdom, contributing to national development and knowledge-based economy goals (Alfaiz et al., 2022).

The Mawhiba Classes Program (MCP) represents one of the foundation’s flagship initiatives, providing self-contained classroom experiences for gifted students across elementary, middle, and high school levels. Students qualify for the program by scoring in the top 2% on the Mawhiba Multiple Cognitive Aptitude Test (MMCAT), a comprehensive assessment developed specifically for the Saudi context (Alharbi and Dimitrov, 2015).

The MMCAT has demonstrated strong psychometric properties, with reliability coefficients ranging from 0.85 to 0.92 across different subtests and age groups (Dimitrov and Alharbi, 2014). The assessment includes measures of verbal reasoning, quantitative reasoning, nonverbal reasoning, and spatial abilities, providing a comprehensive evaluation of cognitive abilities relevant to giftedness identification (National Center for Assessment, 2018).

### Rationale and significance of the study

While the MCP has been operating for 15 years, systematic evaluation of student satisfaction with the program has been limited. Understanding student perspectives is crucial for program improvement and ensuring that the substantial investment in gifted education is meeting its intended goals. Student satisfaction data can inform decisions about program design, teacher training, resource allocation, and policy development.

This study addresses a significant gap in the literature by providing comprehensive assessment of student satisfaction with a large-scale gifted education program in a non-Western context. The findings contribute to international understanding of gifted education program evaluation and provide insights relevant to other educational systems implementing similar programs.

### Purpose and research questions

The purpose of this study was to assess student satisfaction with the MCP across multiple dimensions and to examine how satisfaction varies by gender and educational level. Specifically, the study addressed the following research questions:

1. Are there significant differences in satisfaction levels between male and female students?
2. Do satisfaction levels vary significantly across elementary, middle, and high school levels?

## Method

### Research design

This study employed a quantitative design to assess student satisfaction with the MCP. The quantitative approach was selected to enable systematic measurement of satisfaction across multiple dimensions and to facilitate statistical analysis of group differences and relationships between variables (Creswell and Creswell, 2018).

### Participants

The study included 1,403 students enrolled in the MCP across Saudi Arabia during the 2023–2024 academic year. The sample represented approximately 18% of the total population in the MCP. Participants were selected through stratified random sampling to ensure representation across gender, educational level, and geographic region. The sampling frame included all students enrolled in the MCP during the data collection period, with stratification ensuring proportional representation across key demographic variables.

The final sample consisted of 753 male students (53.7%) and 650 female students (46.3.0%), demonstrating balanced gender representation. Educational level distribution was equally balanced with 162 elementary students (grades 4–6, 11.5%), 551 middle school students (grades 7–9, 39.3%), and 690 high school students (grades 10–12, 49.2%). Participant ages ranged from 9 to 18 years with a mean age of 13.2 years (SD = 2.8). Male students had a mean age of 13.1 years (SD = 2.7, range = 9–18 years), while female students had a mean age of 13.3 years (SD = 2.9, range = 9–18 years).

### How are schools selected?

Schools were selected based on eight criteria established by Mawhiba to ensure program quality and consistency. These criteria included administrative support with strong leadership commitment to gifted education, teacher qualifications requiring certified teachers with gifted education training, appropriate physical infrastructure and learning resources, adherence to MCP curriculum standards, availability of student support services including counseling and guidance, active family involvement programs, regular monitoring of student progress through established assessment practices, and ongoing professional development opportunities for teachers. All participating schools met these criteria as verified through Mawhiba’s quality assurance process.

### Instrumentation

A comprehensive questionnaire was developed to assess student satisfaction with the MCP. The instrument development process was systematic and rigorous, beginning with an extensive literature review of student satisfaction instruments in gifted education contexts. An expert panel consisting of five specialists in gifted education and educational measurement reviewed the initial item pool, providing feedback on content validity, item clarity, and cultural appropriateness for the Saudi context.

Following expert review, the instrument underwent pilot testing with 150 students who were not included in the main study. Based on pilot testing results, items were refined to improve clarity and eliminate any problematic items. The final questionnaire included 30 items distributed across three dimensions.

An EFA was conducted to explore the underlying factor structure of the 30 items. The Kaiser-Meyer-Olkin (KMO) measure of sampling adequacy was 0.98, indicating that the data were suitable for factor analysis. Bartlett’s test of sphericity was significant, *χ*^2^(435) = 35945.12, *p* < 0.001, supporting the factorability of the correlation matrix. We used principal component analysis with Promax rotation to extract the factors. The analysis yielded a three-factor solution that explained 68.24% of the total variance. The factors corresponded to the hypothesized dimensions of Registration Procedures, Learning and Teaching, and Teacher Performance. All items loaded on their respective factors, with loadings ranging from 0.55 to 0.79.

The Registration Procedures dimension consisted of four items assessing satisfaction with program admission, enrollment processes, and initial orientation. A representative item from this dimension was “The registration process for the MCP was clear and easy to understand.” The Learning and Teaching dimension included 14 items evaluating satisfaction with curriculum, instructional methods, and learning activities. The Teacher Performance dimension contained 12 items measuring satisfaction with teacher knowledge, skills, and support.

All items utilized a 5-point Likert scale with response options ranging from 1 (Strongly Disagree) to 5 (Strongly Agree). This scale was selected to provide sufficient response variability while remaining comprehensible for the youngest participants in the study.

Validity evidence for the questionnaire was established through multiple approaches. Face validity was confirmed through expert panel review, with all experts agreeing that items appropriately represented the intended constructs and were suitable for the target population. Content validity was assessed using content validity ratios (CVR), with all items exceeding the minimum threshold of 0.62 for five experts, indicating adequate content representation.

Reliability analysis demonstrated strong internal consistency across all dimensions. The Registration Procedures dimension achieved a Cronbach’s alpha of 0.87, the Learning and Teaching dimension was = 0.91, the Teacher Performance dimension was = 0.89. The total scale reliability for the 30 items was = 0.93.

### Data collection procedures

Data collection was conducted in the last week of second semester in 2024. The study was coordinated with Mawhiba, which oversaw the program implementation. To ensure data quality and minimize potential bias, questionnaires were administered by research assistants. This approach was designed to create neutral administration conditions and reduce the potential for teacher influence on student responses. Research assistants provided standardized instructions to all participants, emphasizing the confidentiality of responses and the importance of honest feedback for program improvement. All questionnaires contained identical item statements. To ensure comprehension among elementary school participants, research assistants administered the questionnaire items orally, reading each statement aloud to participants. Students completed questionnaires electronically.

### Data analysis

Prior to analysis, data were screened for accuracy, missing values, and statistical assumptions. Descriptive analysis included calculation of means and standard deviations. One-way analysis of variance (ANOVA) was used to examine differences across educational levels. Post-hoc comparisons using Bonferroni correction were conducted to identify specific group differences.

## Results

*Q1*: Are there significant differences in satisfaction levels between male and female students?

The analysis of students’ satisfaction on the MCP based on gender showed minimal differences (Table 1). Both male and female students rated the Average Registration dimension equally (*M* = 4.23), indicating high satisfaction. Females slightly outscored males in the Average Learning and Teaching dimension (*M* = 4.12 vs. 4.09), while males rated the Average Teacher Performance dimension higher (*M* = 4.22 vs. 4.13). Statistical tests confirmed these differences were not significant. ANOVA results showed no significant gender effect (*F* = 1.85, *p* = 0.174), and Bonferroni post-hoc tests revealed no meaningful differences across dimensions. Students’ attitudes toward the MCP were positive and did not significantly differ by gender, with both male and female students reporting similarly high levels of satisfaction.

**Table 1:** Descriptive analyses of gender based on frequency, mean, and std. deviation.

Item	Gender	Freq.	*M*	*SD*
The registration steps for Mawhiba classes were clear and easy. The registration steps for Mawhiba classes were clear and easy.	M	753	4.41	0.79
	F	650	4.36	0.78
The information and goals of the program on the website were clear.	M	753	4.26	0.91
	F	650	4.26	0.88
The method of communication from Mawhiba with me before the start of the program was suitable.	M	753	4.19	0.99
	F	650	4.22	0.89
My inquiries were answered before the start of the program.	M	753	4.06	1.03
	F	650	4.07	0.99
**Average Registration Procedures Dimension**	**M**	**753**	**4.23**	**0.76**
	**F**	**650**	**4.23**	**0.73**
The program enhanced my understanding of fundamental concepts and theories in science, mathematics, and technology.	M	753	4.15	1.03
	F	650	4.09	1
The program contributed to developing my critical thinking and problem-solving abilities.	M	753	4.13	1.06
	F	650	4.16	0.97
The program enhanced my ability for self-learning (the ability to learn independently with minimal assistance).	M	753	4.12	1.06
	F	650	4.19	1.01
The program taught me methods and ways to acquire new knowledge.	M	753	4.15	1.03
	F	650	4.22	0.96
The program enhanced my ability to work collaboratively.	M	753	4.15	1.07
	F	650	4.23	0.94
The program contributed to developing my skills in expressing ideas both orally and in writing.	M	753	4.01	1.12
	F	650	4.12	0.97
The program contributed to the development of my personal skills.	M	753	4.12	1.04
	F	650	4.08	1.05
The program provided me with knowledge of new facts and terms in the scientific field.	M	753	4.27	0.93
	F	650	4.28	0.91
I found the activities and learning tools used in the program to be diverse and engaging.	M	753	3.93	1.16
	F	650	3.9	1.15
The program’s approach contributed to developing my skills in exploration, deduction, and information seeking.	M	753	4.14	1.01
	F	650	4.22	0.96
The program helped me identify the scientific field that matches my abilities and interests.	M	753	3.9	1.18
	F	650	3.93	1.12
The program helped me improve my school achievements.	M	753	4.06	1.11
	F	650	4.07	1.1
The program improved my skills in using technological tools and conducting online research.	M	753	4.02	1.12
	F	650	4.25	0.99
The Mawhiba classes environment was stimulating and encouraging for learning.	M	753	4.08	1.13
	F	650	3.97	1.19
**Average Learning & Teaching Dimension**	**M**	**4.09**	**0.88**	**753**
	**F**	**4.12**	**0.83**	**650**
The teacher effectively utilized the lesson time.	M	753	4.23	1.02
	F	650	4.06	1.08
The teacher demonstrated mastery of the subject matter and helped me execute the activities with ease and clarity.	M	753	4.29	0.98
	F	650	4.1	1.05
The teacher is well-prepared for the lessons.	M	753	4.35	0.95
	F	650	4.12	1.07
The teacher progresses through the lesson at a pace suitable for the students’ level.	M	753	4.15	1.09
	F	650	4.01	1.13
The teacher respects the students and answers their questions.	M	753	4.41	0.91
	F	650	4.37	0.88
The teacher presents the information in an engaging manner.	M	753	4.14	1.05
	F	650	3.95	1.12
The teacher encourages students to engage in self-learning and achieve challenging goals.	M	753	4.24	1.01
	F	650	4.23	1.01
The teacher possesses a high ability to integrate technology into teaching.	M	753	4.06	1.11
	F	650	4.1	1.07
The teacher answered our questions in a timely manner and provided feedback.	M	753	4.27	0.98
	F	650	4.21	0.97
The teacher connects the subject topics to practical life.	M	753	4.26	0.96
	F	650	4.23	1
The teacher identifies the appropriate place in the official curriculum content to present Mawhiba activities before the class time.	M	753	4.02	1.19
	F	650	3.94	1.18
The teacher informs us in advance about the times for implementing Mawhiba activities in the classroom.	M	753	4.21	1.08
	F	650	4.19	1
**Average Teacher Performance Dimension**	**M**	**753**	**4.22**	**0.86**
	**F**	**650**	**4.13**	**0.87**

Bold values: The total sum of scores for each dimension was calculated and averaged.

*Q2*: Do satisfaction levels vary significantly across elementary, middle, and high school levels?

The analysis of students’ satisfaction toward the MCP across different school levels (high school, middle school, and elementary school) revealed significant differences in perceptions (Table 2). Descriptive statistics showed that elementary school students consistently reported the highest levels of satisfaction across all dimensions, with a total mean score of 4.38, compared to 4.22 for middle school students and 4.15 for high school students. Specifically, elementary students scored the highest in the Average Registration dimension (*M* = 4.32), Average Learning & Teaching dimension (*M* = 4.28), and Average Teacher Performance dimension (*M* = 4.36). Middle school and high school students also rated these dimensions highly, but with slightly lower mean scores.

**Table 2:** Descriptive analyses of school level based on frequency, mean, and std. deviation.

Item	SL	Freq.	M	*SD*
The registration steps for Mawhiba classes were clear and easy. The registration steps for Mawhiba classes were clear and easy.	H	690	4.37	0.81
	M	551	4.37	0.77
	E	162	4.49	0.72
The information and goals of the program on the website were clear.	H	690	4.19	0.96
	M	551	4.33	0.85
	E	162	4.36	0.75
The method of communication from Mawhiba with me before the start of the program was suitable.	H	690	4.18	0.96
	M	551	4.21	0.94
	E	162	4.28	0.87
My inquiries were answered before the start of the program.	H	690	3.99	1.07
	M	551	4.14	0.95
	E	162	4.13	0.95
**Average Registration Procedures Dimension**	**H**	**690**	**4.18**	**0.77**
	**M**	**551**	**4.26**	**0.74**
	**E**	**162**	**4.32**	**0.67**
The program enhanced my understanding of fundamental concepts and theories in science, mathematics, and technology.	H	690	4.03	1.07
	M	551	4.18	0.97
	E	162	4.31	0.92
The program contributed to developing my critical thinking and problem-solving abilities.	H	690	4.08	1.06
	M	551	4.17	0.98
	E	162	4.32	0.91
The program enhanced my ability for self-learning (the ability to learn independently with minimal assistance).	H	690	4.12	1.06
	M	551	4.17	1.01
	E	162	4.22	0.99
The program taught me methods and ways to acquire new knowledge.	H	690	4.11	1.06
	M	551	4.24	0.94
	E	162	4.33	0.89
The program enhanced my ability to work collaboratively.	H	690	4.17	1.05
	M	551	4.17	1
	E	162	4.35	0.87
The program contributed to developing my skills in expressing ideas both orally and in writing.	H	690	3.95	1.11
	M	551	4.12	1.01
	E	162	4.3	0.9
The program contributed to the development of my personal skills.	H	690	4.02	1.1
	M	551	4.15	1.01
	E	162	4.31	0.89
The program provided me with knowledge of new facts and terms in the scientific field.	H	690	4.19	0.99
	M	551	4.34	0.85
	E	162	4.4	0.82
I found the activities and learning tools used in the program to be diverse and engaging.	H	690	3.85	1.2
	M	551	3.93	1.14
	E	162	4.16	1.01
The program’s approach contributed to developing my skills in exploration, deduction, and information seeking.	H	690	4.14	1.01
	M	551	4.19	0.99
	E	162	4.27	0.9
The program helped me identify the scientific field that matches my abilities and interests.	H	690	3.79	1.21
	M	551	4	1.1
	E	162	4.15	1.03
The program helped me improve my school achievements.	H	690	3.96	1.19
	M	551	4.14	1.04
	E	162	4.28	0.89
The program improved my skills in using technological tools and conducting online research.	H	690	4.13	1.08
	M	551	4.09	1.1
	E	162	4.24	0.91
The Mawhiba classes environment was stimulating and encouraging for learning.	H	690	4.01	1.17
	M	551	3.97	1.18
	E	162	4.33	1
**Average Learning & Teaching Dimension**	**H**	**690**	**4.04**	**0.89**
	**M**	**551**	**4.13**	**0.83**
	**E**	**162**	**4.28**	**0.78**
The teacher effectively utilized the lesson time.	H	690	4.1	1.07
	M	551	4.15	1.05
	E	162	4.36	0.92
The teacher demonstrated mastery of the subject matter and helped me execute the activities with ease and clarity.	H	690	4.14	1.06
	M	551	4.24	0.98
	E	162	4.35	0.93
The teacher is well-prepared for the lessons.	H	690	4.19	1.03
	M	551	4.25	1.04
	E	162	4.45	0.81
The teacher progresses through the lesson at a pace suitable for the students’ level.	H	690	4.04	1.14
	M	551	4.07	1.12
	E	162	4.35	0.9
The teacher respects the students and answers their questions.	H	690	4.4	0.88
	M	551	4.36	0.93
	E	162	4.44	0.83
The teacher presents the information in an engaging manner.	H	690	3.99	1.1
	M	551	4.06	1.11
	E	162	4.3	0.91
The teacher encourages students to engage in self-learning and achieve challenging goals.	H	690	4.21	1.01
	M	551	4.21	1.04
	E	162	4.38	0.89
The teacher possesses a high ability to integrate technology into teaching.	H	690	4.1	1.09
	M	551	3.99	1.15
	E	162	4.32	0.83
The teacher answered our questions in a timely manner and provided feedback.	H	690	4.24	0.98
	M	551	4.22	0.98
	E	162	4.32	0.94
The teacher connects the subject topics to practical life.	H	690	4.23	0.99
	M	551	4.21	1.01
	E	162	4.41	0.82
The teacher identifies the appropriate place in the official curriculum content to present Mawhiba activities before the class time.	H	690	3.96	1.22
	M	551	3.95	1.19
	E	162	4.2	1.03
The teacher informs us in advance about the times for implementing Mawhiba activities in the classroom.	H	690	4.16	1.08
	M	551	4.19	1.02
	E	162	4.4	0.9
**Average Teacher Performance Dimension**	**H**	**690**	**4.15**	**0.87**
	**M**	**551**	**4.16**	**0.88**
	**E**	**162**	**4.36**	**0.76**

SL, School Level; H, high school; M, Middle School; E, elementary school.

Bold values: The total sum of scores for each dimension was calculated and averaged.

The results of the ANOVA confirmed these differences were statistically significant. The effect of school level was significant (*F* = 6.94, *p* = 0.001, η^2^ = 0.01). However, the corresponding effect size was small (η^2^ = 0.01), indicating that only 1% of the total variance in satisfaction scores is attributable to the students’ school level. Post-hoc Bonferroni tests further revealed that elementary school students had significantly higher scores compared to both high school students (mean difference = 0.23, *p* = 0.001) and middle school students (mean difference = 0.16, *p* = 0.042). However, the difference between high school and middle school students was not statistically significant (mean difference = 0.07, *p* = 0.248).

## Discussion

The results demonstrated that students participating in the MCP exhibit very high levels of satisfaction. This positive finding indicates that the program succeeds in meeting the educational needs and expectations of its participants. This conclusion aligns with theoretical frameworks that emphasize the importance of providing specialized educational environments for gifted learners, where appropriate academic challenge, specialized instruction, and opportunities for interaction with similarly capable peers are fundamental factors for their satisfaction (Renzulli, 2012; VanTassel-Baska, 2018).

The high levels of satisfaction across all program dimensions indicate a comprehensive and integrated approach to program design and implementation. This holistic success is particularly noteworthy when considering the complexities associated with implementing large-scale gifted education programs and the diversity in the needs of this student population (Subotnik et al., 2011).

One of the most notable findings of this study was the absence of statistically significant differences in satisfaction between male and female students. Although minor differences in mean scores existed between genders across some dimensions, they were not practically meaningful, suggesting that the program provides a positive and equitable educational experience for both genders. This finding holds particular significance in the Saudi cultural context, where gender disparities in education have been a historical issue, reflecting the program’s success in achieving a high degree of inclusivity and gender equity.

These results differ from some previous research that documented larger gaps in educational satisfaction between genders (Preckel et al., 2008). The convergence in satisfaction levels observed in this study may reflect the efforts made by the MCP to create supportive and inclusive learning environments that meet the needs of all students, regardless of gender.

The analysis of student satisfaction across different school levels revealed a statistically significant, albeit small, trend of declining satisfaction with age. This represents a key finding for program evaluation and continuous improvement. Descriptive statistics showed that elementary school students reported the highest level of satisfaction, followed by middle school students, and then high school students. However, the small effect size suggested that while the trend is real and not due to chance, school level is not a primary driver of student satisfaction.

These results align with developmental literature suggesting that student satisfaction can decrease during adolescence (Neihart, 2007). One possible interpretation is that as students mature, their expectations become more sophisticated, leading them to evaluate educational experiences more critically (Margot and Melin, 2021). Another contributing factor could be the changing priorities of adolescents, with high school students placing a greater emphasis on college and career preparation, which may not align as closely with the current program structure (Olszewski-Kubilius and Clarenbach, 2012). Social factors, such as the importance of peer relationships, might also influence how older students perceive specialized programs (Peterson, 2009).

These findings suggest that a developmentally-sensitive approach to program design could be beneficial. While the difference between middle and high school students was not significant, the overall downward trend suggests a need to consider the unique needs of older students. Incorporating more student choice, career-focused content, and opportunities for autonomy in the high school curriculum could be explored as potential strategies to enhance engagement and satisfaction.

The high satisfaction levels might be interpreted within the Saudi Arabian cultural and educational context. It is possible that the cultural value placed on education and specialized programs contributes to positive student attitudes. The significant investment in gifted education by Mawhiba could also be a factor in creating a high-quality program environment. Similarly, the minimal gender differences in satisfaction could be viewed as a positive indicator of an inclusive program environment.

### Theoretical contributions

This study contributes to the theoretical understanding of student satisfaction in gifted education by providing empirical evidence for the multidimensional nature of satisfaction and the relative importance of different program components. The findings support self-determination theory by demonstrating that satisfaction is enhanced when programs address multiple aspects of the educational experience, including competence (through appropriate challenge), autonomy (through specialized programming), and relatedness (through peer interaction).

The developmental patterns observed support expectancy-value theory by showing how changing expectations and values across age groups influence satisfaction with educational programs. The declining satisfaction with age suggests that programs must evolve to meet changing student needs and expectations.

### Limitations

Several limitations should be acknowledged when interpreting the findings of this study: (a) the study provides a snapshot of student satisfaction at one point in time and cannot establish causal relationships or track changes in satisfaction over time, (b) satisfaction measures rely on student self-reports, which may be influenced by social desirability bias or cultural factors affecting response patterns, and (c) the findings are specific to the Saudi Arabian context and may not be generalizable to other cultural or educational settings, (d) the study examines one specific program model and the findings may not be generalizable to other approaches to gifted education, (e) the study focuses on satisfaction rather than other outcomes such as academic achievement, creativity development, or long-term educational and career outcomes.

## Conclusion

This study provides comprehensive evidence that students in the MCP report high levels of satisfaction across multiple dimensions of their educational experience. The findings demonstrate that the program is successfully meeting many of the educational needs and expectations of gifted students in the Saudi Arabian context.

The significant decline in satisfaction from elementary to high school levels represents the most important finding for program improvement. This pattern suggests that while the program is highly successful with younger students, there is substantial opportunity for enhancement in serving older students. The developmental considerations highlighted by this finding should inform future program modifications and improvements.

As educational systems worldwide continue to invest in gifted education programs, the systematic assessment of student satisfaction provides valuable insights for program improvement and accountability. The methods and findings of this study offer a framework for similar evaluations in other contexts and contribute to the evidence base for effective gifted education practices.

The high satisfaction levels observed in this study, combined with the identification of specific areas for improvement, provide a strong foundation for the continued development of the MCP. The findings support the program’s overall effectiveness while highlighting specific opportunities for enhancement that could further improve student experiences and outcomes.

Future research building on these findings, particularly longitudinal studies and mixed-method approaches, will provide additional insights into the factors that contribute to successful gifted education programs and the long-term impacts of student satisfaction on educational and career outcomes. Such research will continue to inform evidence-based practices in gifted education and support the development of programs that effectively serve the unique needs of gifted learners.

## Data Availability

The raw data supporting the conclusions of this article will be made available by the authors, without undue reservation.
